# The Relationship of 25(OH)D_3_ with Diabetes Mellitus and the Mediation Effect of Lipid Profile in Chinese Rural Population of Henan Province

**DOI:** 10.3390/medicina58010085

**Published:** 2022-01-06

**Authors:** Mimi Zhang, Fei Yu, Yuan Xue, Lulu Song, Mengsi Du, Xing Li, Wenjie Li

**Affiliations:** College of Public Health, Zhengzhou University, Zhengzhou 450001, China; zhangmimi@gs.zzu.edu.cn (M.Z.); xueyuansnow@zzu.edu.cn (Y.X.); 202012272014867@gs.zzu.edu.cn (L.S.); 202022272014879@gs.zzu.edu.cn (M.D.); lwj@zzu.edu.cn (W.L.)

**Keywords:** vitamin D, serum 25(OH)D_3_ level, serum lipid profile, dyslipidemia, T2DM

## Abstract

*Background and Objectives*: Studies suggest that vitamin D is involved in the development of type 2 diabetes mellitus (T2DM) and influences serum lipids levels, while lipid disorders are also closely related to T2DM. This study attempts to explore the complex relationship of serum 25(OH)D_3_, serum lipids, and T2DM among Chinese population. *Materials and Methods*: A cross-sectional study was carried out among 2326 subjects. The chi-square (*χ*^2^) test was applied to compare the prevalence of T2DM or dyslipidemia between two serum 25(OH)D_3_ levels. Linear regression was applied to analyze the correlation between serum lipids and 25(OH)D_3_ contents. Univariate and logistic analysis were used to explore the relationship between two lipid levels and T2DM. Mediation analysis was used to explore whether serum lipids mediate the relationship between two serum 25(OH)D_3_ levels and T2DM. *Results*: Compared to subjects with 25(OH)D_3_ ≥ 30 ng/mL, subjects with 25(OH)D_3_ < 30 ng/mL were higher in the prevalence of T2DM. The occurrences of high TG and low HDL-C were significantly higher in vitamin D deficiency and insufficiency than those in vitamin D sufficiency. Serum 25(OH)D_3_ content showed a reverse correlation with TC, TG, and LDL-C, but positive correlation with HDL-C. The odds ratios (ORs) (95% confidence intervals, 95%CI) of T2DM by comparing TG ≥ 2.26 mmol/L vs. TG < 2.26 mmol/L and HDL-C < 1.04 mmol/L vs. HDL-C ≥ 1.04 mmol/L in all participants were 2.48 (1.94–3.18) and 1.37 (1.07–1.75), respectively. Serum TG or HDL-C level partially mediated the relationship between two 25(OH)D_3_ level and T2DM. *Conclusions*: Serum 25(OH)D_3_ < 30 ng/mL seems to be associated with T2DM or dyslipidemia (high TG and low HDL-C) in our study, but there is still no proof of a cause–effect relationship. Moreover, serum TG or HDL-C level partially mediated the relationship between 25(OH)D_3_ levels and T2DM.

## 1. Introduction

Diabetes mellitus (DM) is a chronic systemic metabolic disease in which the pancreas is incapable of producing enough insulin, or the insulin does not work and cannot send the signal into tissues to consume blood glucose. According to the international diabetes federation (IDF), about 463 million adults worldwide are suffering from diabetes, and China ranks first in the world, which arouses growing attention from a lot of people [1]. It is worth mentioning that type 2 diabetes mellitus (T2DM) accounts for nearly 90% of all diabetes cases [2]. Its clinical manifestations are mainly insufficient insulin secretion and insulin resistance. A long period of hyperglycemia in T2DM patients can eventually cause a series of serious complications [3]. Therefore, early prevention and treatment for T2DM is of great significance, especially as in recent years T2DM has become more frequent in children and adolescents.

At present, the pathogenesis of T2DM is not clear, but it may be related to many aspects, both genetic and environmental. Studies have shown that dyslipidemia is an important hazard factor for a variety of diseases, especially for T2DM [4,5,6]. Dyslipidemia is a chronic abnormal metabolism of lipoproteins, which is closely linked to diet, lifestyle and so on. According to related literature, dyslipidemia is defined as one or more abnormal levels of serum lipids, including total cholesterol (TC), low-density lipoprotein cholesterol (LDL-C), high-density lipoprotein cholesterol (HDL-C), and triglycerides (TG) [7]. Noteworthily, aberrant lipid parameters may be related to insulin resistance and islet β-cell dysfunction [8,9,10]. However, whether dyslipidemia leads to diabetes through causing islet β-cell dysfunction or insulin resistance, the possible pathogenesis has not yet been fully elucidated.

Besides, certain nutrients play a vital role in inducing diabetes. Vitamin D, a vital fat-soluble hormone, has a great influence on regulating bone health, mediating immune function, inflammatory response, and increasing insulin sensitivity, etc. [11,12,13,14]. Vitamin D status in the human body is usually measured by its serum 25(OH)D_3_ level, and defined as: deficient (<20 ng/mL), insufficient (20~30 ng/mL), or adequate (≥30 ng/mL) [15,16,17]. Recently, the high prevalence of vitamin D deficiency and inadequacy has aroused great concern worldwide [18,19]. The correlation between serum 25(OH)D_3_ and lipids has been reported in many studies, but the results are not consistent [20,21]. Moreover, studies have found that vitamin D can improve insulin secretion, insulin sensitivity, and may be equipped with the ability to regulate glucose metabolism [22,23,24,25,26]. Therefore, it could be speculated that vitamin D is closely linked to the process of diabetes. In fact, much research has revealed low contents of vitamin D to be ubiquitous in patients with T2DM [27,28,29]. Vitamin D supplementation relates to a lower risk of T2DM, whereas vitamin D deficiency and insufficiency may promote the occurrence of T2DM [30]. However, several studies have also shown that no link could be established between vitamin D and T2DM [31,32].

All in all, the relationships among vitamin D, serum lipid profile, and T2DM are uncertain and still need further exploring. Therefore, this article adopted a field investigation to initially explore the possible association between serum contents of 25(OH)D_3_, serum lipids, and T2DM among a Chinese rural population. It is expected to provide more theoretical clues and a practical basis for the above issue.

## 2. Materials and Methods

### 2.1. Ethics Statement

This proposal was designed according to the Helsinki Declaration and was approved by Zhengzhou University Life Science Ethics Review Committee (2015 MEC (S128), date of approval: 5 March 2015). All participants signed written informed consent.

### 2.2. Selection of Participants

This survey is a cross-sectional study. Participants were enrolled from three different regions, including Zhengzhou, Jiaozuo, and Luoyang City of Henan Province in China from January to May 2013. These individuals complied with the following inclusion criteria: were aged between 2 and 105 years, had no known disease or history of changing vitamin D metabolism, gestational diabetes (GD), and cancer, taken no drugs to control blood lipid, and signed informed consent. Hence, 2378 subjects participated in this study in total and completed several surveys, including questionnaires, collection of blood samples, and detection of serum biomarkers, but only 2326 individuals met the inclusion criteria.

### 2.3. Data Collection

The questionnaire was administered by professional interviewers and contained the information on demographic characteristics, vitamin D supplement and the individual history of illness, such as disease of changing vitamin D metabolism, gestational diabetes mellitus, and cancer, etc.

### 2.4. Blood Samples

To conduct further biochemical analysis on the blood samples, each participant was asked to go on an empty stomach overnight, and then trained nurses collected and tested some indicators the next day. The tested indicators include serum 25(OH)D_3_ levels, lipid profile (TC, TG, LDL-C and HDL-C), fasting blood glucose, and so on.

### 2.5. Laboratory Measurements

The serum 25(OH)D_3_ concentrations were determined using enzyme-linked immunosorbent assay (ELISA) Kits (Sangon Biotech, Shanghai, China). All samples were tested for fasting blood glucose and blood lipid by automatic biochemical analyzer (KHB360, Shanghai, China).

### 2.6. Definition

In this study, vitamin D deficiency was defined as 25(OH)D_3_ < 20 ng/mL. Vitamin D insufficiency was confirmed as 20 ≤ 25(OH)D_3_ < 30 ng/mL. Vitamin D sufficiency was defined as 25(OH)D_3_ ≥ 30 ng/mL [16,33]. According to the definition of WHO in 1999, normal fasting plasma glucose (FPG) is less than 6.1 mmol/ L and impaired fasting glucose (IFG) is between 6.1 and 7.0 mmol/L. For children and adolescents, normal FPG is less than 5.6 mmol/L. IFG is between 5.6 and 6.9 mmol/L [34]. According to WHO guidelines in 1999 and the American Diabetes Association in 2002, we defined T2DM as FPG ≥ 7.0 mmol/L or self-reported history of T2DM diagnosis and taking hypoglycemic therapy after excluding type 1 diabetes, GD and other special types of diabetes. For children and adolescents, the normal cutoff levels were <0.85 mmol/L (0–9 years old) or <1.02 mmol/L (10–18 years old) for TG, <4.4 mmol/L for TC, >1.2 mmoL/L for HDL-C and <2.8 mmol/L for LDL-C, respectively [35,36]. The abnormal thresholds for high TG, high TC, low HDL-C, and high LDL-C were 2.26 mmol/L, 6.22 mmol/L, 1.04 mmol/L, and 4.14 mmol/L, respectively [37,38,39].

### 2.7. Statistical Analysis

For continuous variables with normal distribution, the data were expressed as mean ± standard deviation (SD), and the differences between groups were compared using Student’s t-test and one-way analysis of variance (ANOVA). For categorical variables, the data were represented by percentage or frequency and compared by chi-square test. Logistic regression was used to explore the relationship between T2DM and serum lipid indicators. Linear regression analysis was used to analyze whether 25(OH)D_3_ levels affected serum lipid levels (TC, TG, HDL-C, LDL-C) and fasting glucose levels. Mediation analysis was carried out by using the PROCESS in SPSS 21.0, where the mediator should be continuous variables. This method was used to estimate (1) the total effect of 25(OH)D_3_ levels (X) on T2DM (Y), unadjusted for serum TG or HDL-C (M)—path c; (2) the direct effect of 25(OH)D_3_ levels (X) on T2DM (Y), controlling for the effect of serum TG or HDL-C (M)—path c’; (3) the association between 25(OH)D_3_ levels (X) and the mediator, serum TG or HDL-C (M)—path a; and (4) the association between serum TG or HDL-C (M) and T2DM (Y), controlling for 25(OH)D_3_ levels—path b. Relative effect value was defined as direct effect/total effect or indirect effect/total effect. SPSS 21.0 software package (SPSS Inc., Chicago, IL, USA) was used for all the statistical analyses. Two-sided *p* value < 0.05 was considered to be statistically significant.

## 3. Results

### 3.1. Demography Characteristics of Participants

Table 1 reveals the prevalence of T2DM according to the general demographic characteristics. The study subjects included 1066 males (45.83%) and 1260 females (54.17%). The prevalence rate of T2DM in subjects younger than 18 years old was much lower than that in subjects aged 18 years or older. Average level of serum 25(OH)D_3_ was 33.79 ng/mL. Subjects with T2DM tend to be female farmers and are more likely to have a lower education. Serum 25 (OH) D_3_ levels in people with T2DM were not so high as that in people without T2DM (*p* = 0.001).

### 3.2. Distribution of 25(OH)D_3_ and the Demographic Characteristics

**Figure 1:**
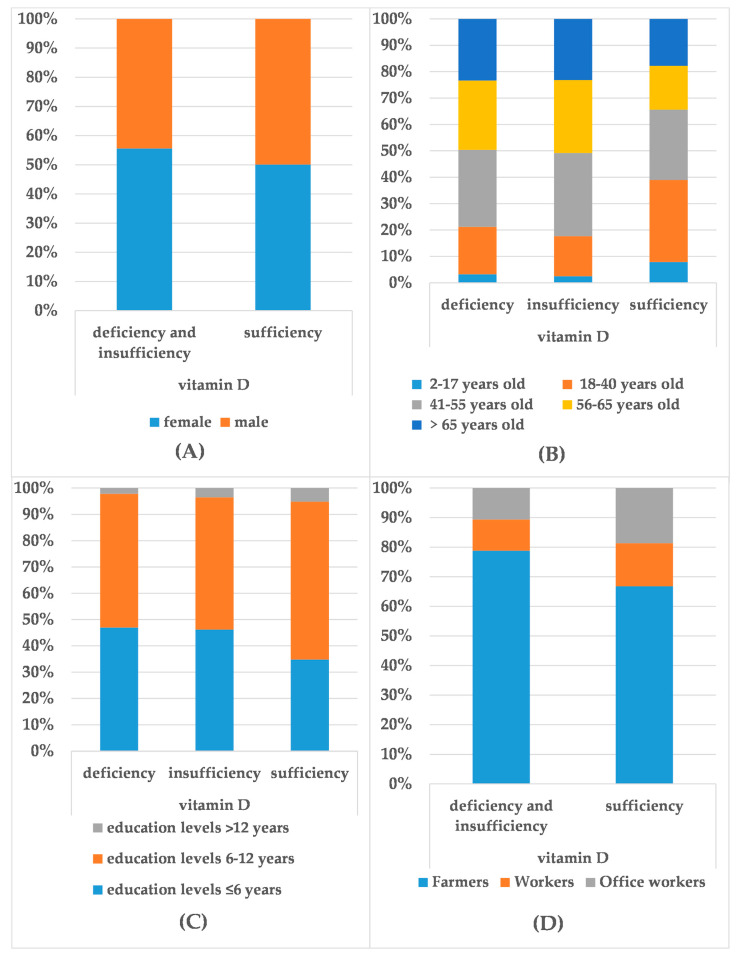
The proportion of the demographic characteristics in different 25(OH)D_3_ levels. The proportion of gender distribution (**A**), Age (years) distribution (**B**), Education (years) levels (**C**), and Occupation distribution (**D**) in different 25(OH)D_3_ levels.

Figure 1 shows the proportional relationships of certain demographic characteristics of participants at different 25(OH)D_3_ levels. As for the incidence of vitamin D deficiency and insufficiency, gender differences can be observed in Figure 1A (*p* = 0.02). Serum 25(OH)D_3_ levels were negatively correlated with participants’ age (r = −0.14, *p* < 0.001) (Figure 1B) and positively correlated with their educational level (r = 0.11, *p* < 0.001) (Figure 1C). In addition, in different occupations, the incidence of vitamin D deficiency and insufficiency in farmers is higher than that in workers (*p* = 0.001, OR = 1.64, 95%CI: 1.24–2.17) and office workers (*p* < 0.001, OR = 2.07, 95%CI: 1.59–2.69) (Figure 1D).

### 3.3. 25(OH)D_3_ Levels, Serum Glucose (GLU) and T2DM

**Figure 2:**
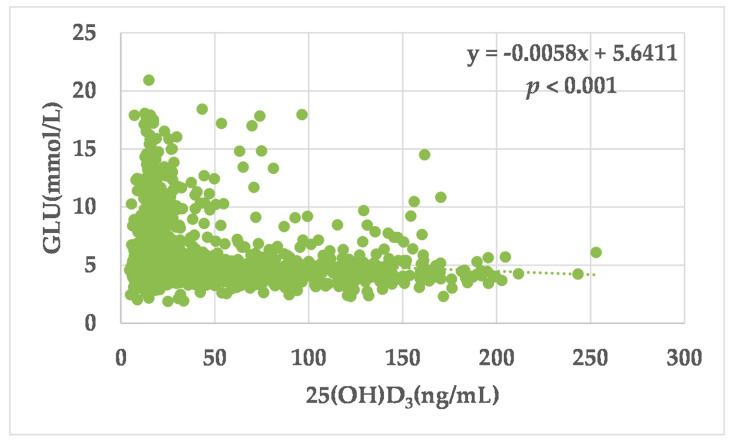
Simple linear correlation between serum GLU and 25(OH)D_3_ level.

A simple linear regression analysis was used to explore the relationship between vitamin D levels and serum glucose (GLU) (Figure 2). GLU showed a significant inverse linear correlation with the level of 25(OH)D_3_ (β coefficient = −0.006, *p* < 0.001).

**Figure 3:**
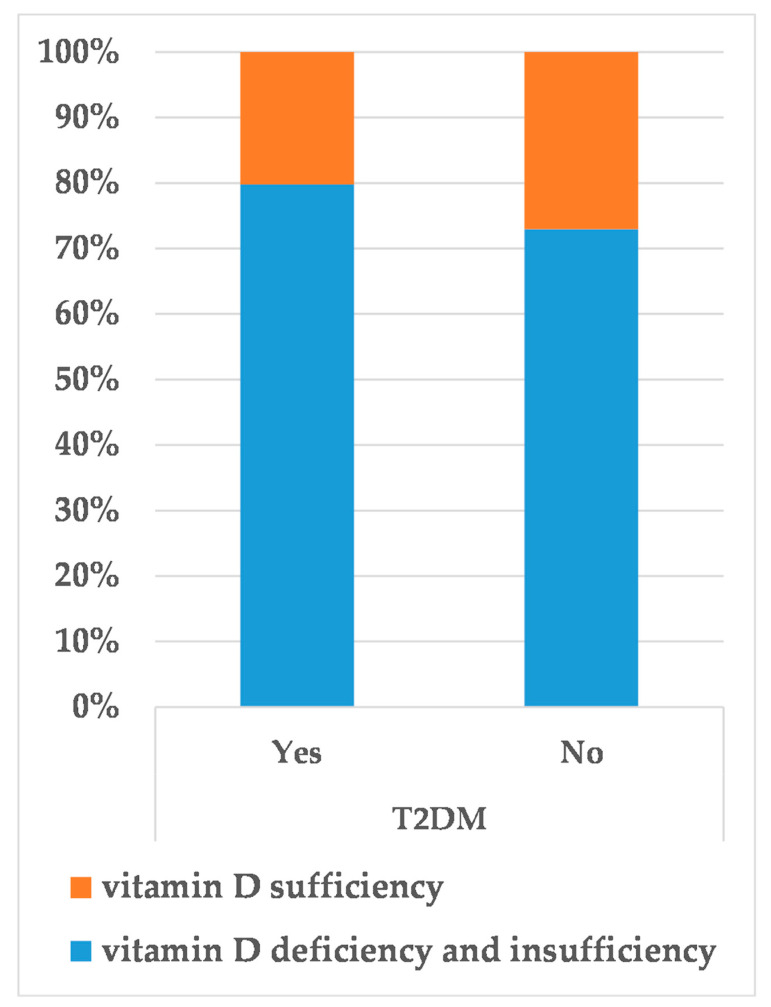
The proportion of T2DM prevalence in different 25(OH)D_3_ levels.

In addition, this study showed that the serum level of 25(OH)D_3_ was associated with the incidence of T2DM, and the prevalence of T2DM in vitamin D deficiency and insufficiency group was much higher than that in vitamin D sufficiency group (*p* = 0.004) (Figure 3).

### 3.4. 25(OH)D_3_ Levels and the Incidence of Dyslipidemias, as Well as Lipid Profile

We divided all participants into two groups based on their serum vitamin D levels: the vitamin D deficiency and insufficiency group and the vitamin D sufficiency group. Differences in the incidence of dyslipidemia were analyzed under these two vitamin D groups. The occurrences of high TG and low HDL-C were significantly higher in vitamin D deficiency and insufficiency group than those in vitamin D sufficiency group (*p* = 0.012 and < 0.001, respectively). However, the difference between the two groups was not statistically significant in terms of the incidence of high TC and high LDL-C (*p* = 0.217 and 0.604, respectively) (Table S1).

**Figure 4:**
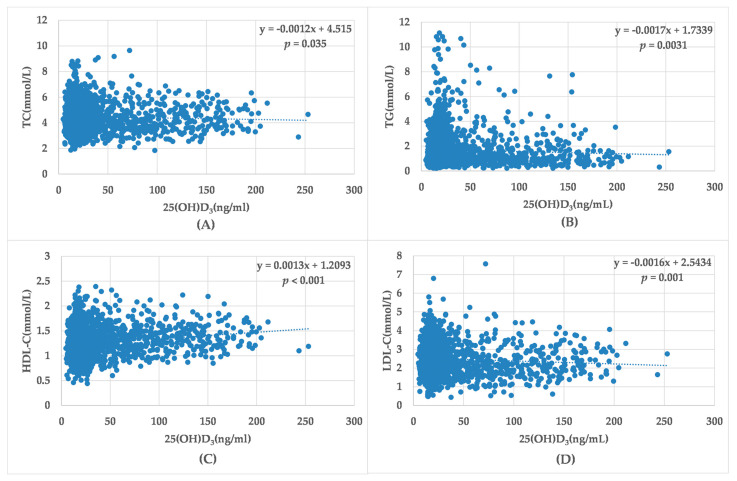
Simple linear correlation between serum lipids and 25(OH)D_3_ level. Simple linear correlation between TC (**A**), TG (**B**), HDL-C (**C**), LDL-C (**D**) and 25(OH)D_3_ level. TC (the cholesterol), TG (triglyceride), HDL-C (high-density lipoprotein- cholesterol), LDL-C (low-density lipoprotein-cholesterol).

A simple linear regression analysis was used to explore the relationship between vitamin D levels and serum lipid components (Figure 4) and to identify potential predictors of low vitamin D levels. Among them, the variables (TC, TG, and LDL-C) showed a significant inverse linear correlation with the level of 25(OH)D_3_: TC (β coefficient = −0.001, *p* = 0.035), TG (β coefficient = −0.002, *p* = 0.031) and LDL-C (β coefficient = −0.002, *p* = 0.001), i.e., the lower the vitamin D level, the higher the level of these factors. However, there was a positive correlation between HDL-C (β coefficient = 0.001, *p* < 0.001) and 25(OH)D_3_ levels.

### 3.5. The Prevalence of T2DM in the Stratified Serum Lipid Profile

As shown in Table S2, people with high TC have a higher risk of developing T2DM than those without high TC (*p* = 0.040). Similarly, the prevalence of T2DM was higher among high TG group (*p* < 0.001) or low HDL-C group (*p* = 0.003) compared with its corresponding control group. No correlation was found between LDL-C levels and T2DM. Moreover, a logistic regression analysis in Table 2 shows that after adjusting for sex, age, education levels, and occupation, subjects with high TG (OR = 2.48, 95%CI: 1.94–3.18, *p* < 0.001) and low HDL-C (OR = 1.37, 95%CI: 1.07–1.75, *p* = 0.011) were found to be linked with the increased risk of T2DM compared with subjects with the other corresponding levels.

### 3.6. 25(OH)D_3_ Level, Serum Lipid Levels (TG or HDL-C) and T2DM

**Figure 5:**
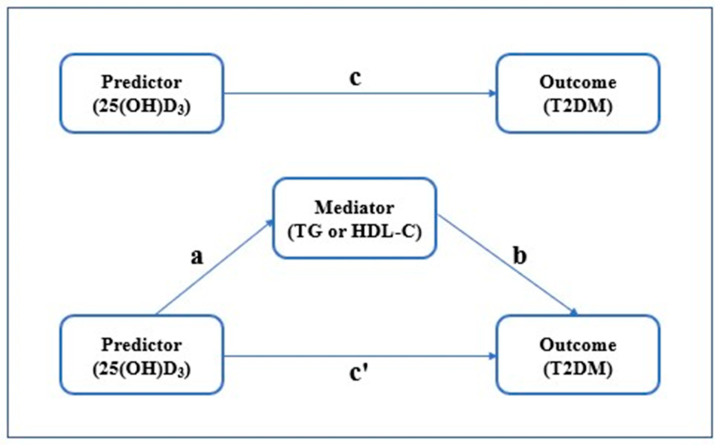
The mediation model: “a” represents the path from 25(OH)D_3_ (Predictor) to TG (Mediator), “b” represents the path from TG or HDL-C (Mediator) to T2DM (Outcome), “c” represents the path from 25(OH)D_3_ (Predictor) to T2DM (Outcome), and “c’” represents the path from 25(OH)D_3_ (Predictor) to T2DM (Outcome) after TG or HDL-C (Mediator) is controlled.

To explore the effect of serum TG or HDL-C level on the association between different 25(OH)D_3_ levels (the vitamin D deficiency and insufficiency group and the vitamin D sufficiency group) and T2DM, we performed a mediation analysis and calculated the proportional effect of serum TG or HDL-C level on this association. Figure 5 displays the model of the mediation effect of TG or HDL-C. The results are presented in Table 3 and Table 4, respectively. In mediation analysis, the estimated OR for T2DM with 25(OH)D_3_ level (total effect, OR = 0.68, 95%CI: 0.51–0.87, *p* = 0.004) included the indirect effect mediated by serum TG (OR = 0.96, 95%CI: 0.91–0.99) or HDL-C (OR = 0.94, 95%CI: 0.89–0.99) and the direct effect of 25(OH)D_3_ level (OR = 0.70, 95%CI: 0.54–0.92, *p* = 0.012 and OR = 0.72, 95%CI: 0.55–0.94, *p* = 0.017, respectively). The result suggested that serum TG or HDL-C level partially mediate the relationship between 25(OH)D_3_ level and T2DM, and the proportion of effect was 10.26% and 15.38%, respectively.

## 4. Discussion

The problem of vitamin D deficiency and insufficiency has received widespread concern around the world. In this study, the mean serum level of 25(OH)D_3_ in all participants was 33.79 ng/mL. Astonishingly, 74.16% of the participants were either vitamin D deficient or insufficient. Of note, serum vitamin D levels were related to the prevalence of T2DM in our study. Compared to individuals with 25(OH)D_3_ ≥ 30 ng/mL, those with 25(OH)D_3_ < 30 ng/mL were associated with a higher risk of T2DM. Consistent with our results, a series of previous studies indicated that low contents of vitamin D were linked with high prevalence of T2DM [39,40,41]. High levels of serum TC, TG, LDL, or low serum content of HDL-C constitute a form of dyslipidemia, which is a common feature and manifestation of dyslipidemia in diabetes [42,43]. In addition, dyslipidemia has also long been considered as a risk factor for T2DM [6,44]. The measurement of some lipids, such as TG, TC and HDL-C, has been used as a predictor of T2DM events [45]. In our study, participants with level of high TG or low HDL-C had an increased risk of T2DM, which is identical with previous conclusions [6,10,44,46]. Moreover, the incidence of high TG and low HDL-C was higher in the vitamin D deficiency and insufficiency group compared with the vitamin D sufficiency group. Furthermore, serum contents of 25(OH)D_3_ were positively related to the serum contents of HDL-C and negatively related to TC, TG, and LDL-C contents among all participants, which is in line with previous studies [21,28,47]. These findings further confirm that low levels of vitamin D may be closely linked to the occurrence of dyslipidemia [20,48]. In addition, there are related researches which pointed out that vitamin D supplementation can improve metabolic indexes, including TC, LDL, HDL, and so on [49]. It was worth noting that serum TG and HDL-C levels were partial mediators of the correlation between serum 25(OH)D_3_ levels and T2DM shown by this study, but the causal relationship between them cannot be proved.

All over the world, there have been extensive observational studies that have explored the correlation with serum vitamin D level and lipid level. For instance, in a study in China, the increase in serum vitamin D3 resulted in an increase in serum HDL-C and decrease in serum TC and LDL-C [20]. In a cross-sectional study of German elderly women aged 66–96 years, when expressed at no less than 62.3 nmol/L, 25(OH)D_3_ was inversely correlated with TC and LDL-C, but positively correlated with HDL-C [50]. In addition, a study conducted in northern Norway found a correlation with serum 25(OH)D and serum lipids. With the increase of serum 25(OH)D level, serum contents of TC, HDL-C, and LDL-C all increased [21]. However, this result was not consistent with the results from the aforementioned cases and our study, possibly due to the difference in study population. Besides the above cross-sectional findings, 25(OH)D has been shown to be positively correlated with HDL and negatively correlated with TG and TC in case-control studies [28]. However, a few experimental epidemiology studies confirmed that vitamin D supplementation has no way to improve serum lipid abnormalities [51,52], or there is no correlation between the two. Therefore, the potential correlation with vitamin D and serum lipids needs to be investigated further. In particular, a large number of interventional studies are needed to explore the possible relationship.

Although the mechanisms of vitamin D affecting lipid levels has not been fully explained, there are several possible explanations worth mentioning. Firstly, vitamin D level can negatively affect parathyroid hormone (PTH) in the human body. A high PTH level may accelerate calcium influx in certain cells, such as adipocytes, thereby increasing lipase expression and then increasing various lipid factors [28,45]. Secondly, vitamin D can restrain the synthesis and excretion of TG by promoting calcium absorption in the intestine. Thirdly, vitamin D may cause an increase in HDL levels by regulating the outflow of cholesterol from cholesterol-carrying macrophages and thus participate in the reverse cholesterol transport process to remove excess cholesterol from the liver [53]. Fourthly, low vitamin D contents may cause cell function damage or insulin hypoglycemic function disorder. The occurrence of these abnormal changes may have adverse effects on the metabolism of lipoprotein, resulting in the decrease of HDL-C content or the increase of TG content. Besides the above indirect impact of vitamin D on the occurrence of dyslipidemia, vitamin D also directly participates in the synthesis of bile acids, improving the conversion rate of cholesterol, thereby regulating the level of cholesterol in the body.

Based on mediation analysis, serum TG or HDL-C level partially mediated the relationship between 25(OH)D_3_ levels and T2DM. Combined with the above analysis on the mechanism of vitamin D and dyslipidemia, the possible mechanism is that the increase of serum TG may cause the increase of free fatty acids and the accumulation of triglycerides in pancreatic β cells [8]. Free fatty acids thereby effectively compete with glucose for the substrates and induce diabetes [54]. In addition, both in vivo and in vitro experiments suggested that continuous exposure of β cells to TG could attenuate the ability of β cells to respond to glucose, and eventually lead to the dysfunction of β cells [55]. In addition, low HDL-C level may predispose to T2DM by damaging the function of β cells and insulin resistance, because a few reports have shown the decrease of HDL-C level is closely linked to the dysfunction of β cells and insulin resistance [56]. At present, the exact mechanism of vitamin D affecting diabetes by restoring serum lipid level has not been clarified, and more research is needed.

This study has some advantages and disadvantages. One of the advantages is that the randomly selected study population includes subjects of a larger age range. Secondly, this study proposed a relatively novel idea that serum TG or HDL-C level might partially mediate the link between 25(OH)D_3_ levels and T2DM. This study also has possible limitations in the following aspects. It is a descriptive study, and the results cannot determine the causal relationship. Further studies, such as intervention studies, are needed to demonstrate this causal effect. Moreover, several vital potential indicators were not included in the current study, such as body mass index (BMI). Obesity is defined as a BMI of 30 kg/m^2^ or more [57]. Notably, studies conducted in most countries and regions around the world have obtained a consistent result: compared with people with normal weight, the serum vitamin D level of obese people was lower and inversely related to BMI [58,59,60]. In fact, it is likely that obesity is an important cause affecting the decrease of serum vitamin D level [61]. The possible reasons for low vitamin D in obese people are insufficient sunlight exposure and volumetric dilution effect, etc. [58]. Therefore, exploring the complex relationship among obesity, vitamin D, and T2DM still needs a host of research, which is worthy of further consideration. Moreover, people of all ages were included, and the study did not specifically focus on the elderly whose vitamin D deficiency and insufficiency are more common. This survey scope focused on only few rural areas, and the conclusions may not be applicable to urban areas.

## 5. Conclusions

Vitamin D deficiency and insufficiency are widespread in China’s rural population, reaching up to 74.16% of the subjects. Vitamin D deficiency and insufficiency may be associated with dyslipidemia (TG ≥ 2.26 mmol/L or HDL-C < 1.04 mmol/L) and T2DM in present study, but a cause–effect relationship has not been proven. Moreover, it is further proved that serum TG or HDL-C level partially mediated the relationship between 25(OH)D_3_ levels and T2DM.

## Figures and Tables

**Table 1:** Characteristics of the study population according to the presence of T2DM.

Variable	Event (T2DM) (*n* = 395)	No Event (*n* = 1931)	*p*
Gender			0.004 *
male	155 (39.24%)	911 (47.18%)	
female	240 (60.76%)	1020 (52.82%)	
Age			<0.001 *
<18	1 (0.25%)	97 (5.02%)	
≥18	394 (99.75%)	1834 (94.98%)	
Education (years)			<0.001 *
≤6	218 (55.10%)	797 (41.30%)	
6~12	173 (43.88%)	1061 (54.94%)	
>12	4 (1.02%)	73 (3.76%)	
Occupation			<0.001 *
Farmer	338 (85.53%)	1424 (73.73%)	
Worker	33 (8.38%)	236 (12.22%)	
Office worker	24 (6.09%)	271 (14.05%)	
GLU (mmol/L)	9.08 ± 3.32	4.71 ± 0.81	<0.001 *
TC (mmol/L)	4.74 ± 1.05	4.42 ± 1.01	<0.001 *
TG (mmol/L)	2.29 ± 1.76	1.55 ± 1.21	<0.001 *
HDL-C (mmol/L)	1.21 ± 0.27	1.26 ± 0.31	0.003 *
LDL-C (mmol/L)	2.56 ± 0.80	2.48 ± 0.78	0.066
25(OH)D_3_ (ng/mL)	29.07 ± 28.76	34.75 ± 37.04	0.001 *

Data are presented as mean ± SD for continuous variables. TC (total cholesterol). TG (triglyceride). HDL-C (high-density lipoprotein cholesterol). LDL-C (low-density lipoprotein cholesterol). 25(OH) D_3_ (25-hydroxyvitamin D_3_). T2DM (type 2 diabetes mellitus); * *p* < 0.05 was considered statistically significant.

**Table 2:** OR and 95%CI for the risk of T2DM according to the levels of serum lipids.

Parameter	Unadjusted			Adjusted		
	OR	95%CI	*p*	OR	95%CI	*p*
TC						
Low TC	1.00 (ref.)			1.00 (ref.)		
High TC	1.58	1.02–2.46	0.040 *	1.34	0.84–2.13	0.220
TG						
Low TG	1.00 (ref.)			1.00 (ref.)		
High TG	2.40	1.89–3.05	<0.001 *	2.48	1.94–3.18	<0.001 *
HDL-C						
High HDL-C	1.00 (ref.)			1.00 (ref.)		
Low HDL-C	1.44	1.14–1.82	0.003 *	1.37	1.07–1.75	0.011 *
LDL-C						
Low LDL-C	1.00 (ref.)			1.00 (ref.)		
High LDL-C	0.65	0.29–1.45	0.295	0.51	0.23–1.15	0.106

Notes: Adjustment for sex, age, education levels and Occupation. OR (odds ratio). CI (confidence interval). TC (total cholesterol). TG (triglyceride). HDL-C (high-density lipoprotein cholesterol). LDL-C (low-density lipoprotein cholesterol); * *p* < 0.05 was considered statistically significant.

**Table 3:** Mediation analysis of the relationship between 25(OH)D_3_ levels and T2DM by TG.

Effects	Parameter Estimate (95%CI)	OR (95%CI)	Relative Effect Value	*p*
Path a	−0.15 (−0.28, −0.02)	0.86 (0.76, 0.98)	—	0.025 *
Path b	0.32 (0.25, 0.39)	1.38 (1.28, 1.48)	—	<0.001 *
Indirect effect-path a, b	−0.04 (−0.09, −0.01)	0.96 (0.91, 0.99)	10.26%	—
Direct effect-path c’	−0.35 (−0.62, −0.08)	0.70 (0.54, 0.92)	89.74%	0.012 *
Total effect-path c	−0.39 (−0.68, −0.14)	0.68 (0.51, 0.87)	100%	0.004 *

Notes: Path a, b coefficients represent 5000 bootstrapped samples and Percentile 95%CIs. OR (odds ratios). CI (confidence interval). TG (triglyceride); * *p* < 0.05 was considered statistically significant.

**Table 4:** Mediation analysis of the relationship between 25(OH)D_3_ levels and T2DM by HDL-C.

Effects	Parameter Estimate (95%CI)	OR (95%CI)	Relative Effect Value	*p*
Path a	0.14 (0.11, 0.16)	1.15 (1.12,1.17)	—	<0.001 *
Path b	−0.47 (−0.84, −0.10)	0.63 (0.43,0.90)	—	0.013 *
Indirect effect-path a, b	−0.06 (−0.12, −0.01)	0.94 (0.89,0.99)	15.38%	—
Direct effect-path c’	−0.33 (−0.60, −0.06)	0.72 (0.55,0.94)	84.62%	0.017 *
Total effect-path c	−0.39 (−0.68, −0.14)	0.68 (0.51,0.87)	100%	0.004 *

Notes: Path a, b coefficients represent 5000 bootstrapped samples and Percentile 95%CIs. OR (odds ratios). CI (confidence interval). HDL-C (high-density lipoprotein cholesterol); * *p* < 0.05 was considered statistically significant.

## Data Availability

The datasets involved in this article can be obtained from the corresponding author according to reasonable requirements.

## Supplementary Materials

The following are available online at https://www.mdpi.com/article/10.3390/medicina58010085/s1, Table S1: The Comparison of serum lipid characteristics under different 25(OH)D_3_ levels, Table S2: The Comparison of the prevalence of T2DM in different serum lipid levels.
