# Supplementary material for: The Relationship of 25(OH)D3 with Diabetes Mellitus and the Mediation Effect of Lipid Profile in Chinese Rural Population of Henan Province
Source: Medicina (Kaunas). 2022 Jan 6;58(1):85. doi: 10.3390/medicina58010085 (PMC8781849; doi:10.3390/medicina58010085)
Supplement: Supplementary file 1 [file medicina-58-00085-s001.zip › medicina-1527729-supplementary.pdf]

Supplementary Materials

Table S1. The Comparison of serum lipid characteristics under different 25(OH)D_3 levels.

**Variable**	**vitamin D deficiency and insufficiency (N=1725)**	**vitamin D sufficiency (N= 601)**	**χ^2-value**	***p***
High TC	4.71%	5.99%	1.53	0.217
High TG	21.76%	16.95%	6.28	0.012*
Low HDL-C	30.08%	13.29%	65.76	<0.001*
High LDL-C	2.57%	2.97%	0.27	0.604

OR (odds ratio). CI (confidence interval). TC (total cholesterol). TG (triglyceride). HDL-C (high-densit y lipoprotein cholesterol). LDL-C (low-density lipoprotein cholesterol); Data are presented as percentage of each category. * $p< 0.05$ was considered statistically significant.

Table S2. The Comparison of the prevalence of T2DM in different serum lipid levels.

Parameter	Event(T2DM) (N=395)	No event (N=1931)	χ^2-value	p
**TC**			4.22	0.040*
Low TC	16.6%	83.4%		
High TC	23.9%	76.1%		
**TG**			54.54	<0.001*
Low TG	14.1%	85.9%		
High TG	28.3%	71.7%		
**HDL-C**			8.64	0.003*
High HDL-C	15.6%	84.4%		
Low HDL-C	20.9%	79.1%		
**LDL-C**			1.13	0.289
Low LDL-C	17.1%	82.9%		
High LDL-C	11.9%	88.1%		

OR (odds ratio). CI (confidence interval). TC (total cholesterol). TG (triglyceride). HDL-C (high-density lipoprotein cholesterol). LDL-C (low-density lipoprotein cholesterol); Data are presented as percentage of each category. * $p < 0.05$ was considered statistically significant.
